# Acute effects of glucagon‐like peptide‐1, GLP‐1_9–36 amide_, and exenatide on mesenteric blood flow, cardiovascular parameters, and biomarkers in healthy volunteers

**DOI:** 10.14814/phy2.13102

**Published:** 2017-02-24

**Authors:** Lasse Bremholm, Ulrik B. Andersen, Mads Hornum, Linda Hilsted, Simon Veedfald, Bolette Hartmann, Jens Juul Holst

**Affiliations:** ^1^ Department of Medicine (Gastroenterology Section)Koege Hospital, University of CopenhagenCopenhagenDenmark; ^2^ Department of Clinical Physiology and Nuclear Medicine and PETRigshospitalet (Glostrup Section)University of CopenhagenCopenhagenDenmark; ^3^ Department of NephrologyRigshospitaletUniversity of CopenhagenCopenhagenDenmark; ^4^ Department of Clinical BiochemistryRigshospitaletUniversity of CopenhagenCopenhagenDenmark; ^5^ Department of Biomedical Sciences & NNF Center for Basic Metabolic ResearchThe Panum InstituteUniversity of CopenhagenCopenhagenDenmark

**Keywords:** Exenatide, glucagon like‐peptide‐1, mesenteric blood flow

## Abstract

Glucagon‐like peptide‐1 (GLP‐1, GLP‐1_7–36amide_) and its sister peptide glucagon‐like peptide 2 (GLP‐2) influence numerous intestinal functions and GLP‐2 greatly increases intestinal blood flow. We hypothesized that GLP‐1 also stimulates intestinal blood flow and that this would impact on the overall digestive and cardiovascular effects of the hormone. To investigate the influence of GLP‐1 receptor agonism on mesenteric and renal blood flow and cardiovascular parameters, we carried out a double‐blinded randomized clinical trial. A total of eight healthy volunteers received high physiological subcutaneous injections of GLP‐1, GLP‐1_9–36 amide_ (bioactive metabolite), exenatide (stable GLP‐1 agonist), or saline on four separate days. Blood flow in mesenteric, celiac, and renal arteries was measured by Doppler ultrasound. Blood pressure, heart rate, cardiac output, and stroke volume were measured continuously using an integrated system. Plasma was analyzed for glucose, GLP‐1 (intact and total), exenatide and Pancreatic polypeptide (PP), and serum for insulin and C‐peptide. Neither GLP‐1, GLP‐1_9–36 amide_, exenatide nor saline elicited any changes in blood flow parameters in the mesenteric or renal arteries. GLP‐1 significantly increased heart rate (two‐way ANOVA, injection [*P* = 0.0162], time [*P* = 0.0038], and injection × time [*P* = 0.082]; Tukey post hoc GLP‐1 vs. saline and GLP‐1_9–36amide_ [*P* < 0.011]), and tended to increase cardiac output and decrease stroke volume compared to GLP‐1_9–36 amide_ and saline. Blood pressures were not affected. As expected, glucose levels fell and insulin secretion increased after infusion of both GLP‐1 and exenatide.

## Introduction

The hormone glucagon‐like peptide‐1 (GLP‐1, GLP‐1_7–36amide_) is best known for its actions on the endocrine pancreas, where it potentiates glucose‐induced insulin secretion via activation of specific GLP‐1 receptors and inhibits glucagon secretion (Holst 2007). These actions led to the development of GLP‐1 receptor agonists (GLP‐1‐RAs), which are now being used worldwide for the treatment of type 2 diabetes (Holst et al. 2008). But GLP‐1 has other activities, which are important both physiologically and therapeutically. Thus, it inhibits appetite, and thereby food intake (Flint et al. 1998), which is expedient considering the association between obesity and type 2 diabetes, and a GLP‐1 receptor agonist has recently been approved for the therapy of obesity (Pi‐Sunyer et al. 2015). Cardiovascular actions of GLP‐1 and the GLP‐1 RA have also attracted considerable attention (Sivertsen et al. 2012) because the agonists: (1) may improve myocardial performance; (2) may reduce myocardial damage after ischemia; (3) may improve endothelial dysfunction in diabetes; and (4) clearly improve cardiovascular risk factors, including improved blood pressure, lowered body weight, improved blood lipids, and beneficial changes in high‐sensitivity C‐reactive protein (hsCRP), plasminogen activator inhibitor‐1 (PAI‐1), and (pro)brain natriuretic peptide ((pro)BNP) (Tate et al. 2015). The mechanisms behind these actions are unclear; some may be related to the general metabolic improvement obtained during GLP‐1 therapy (weight loss, improved glycemic control); others may be related to interaction with GLP‐1 receptors in the involved organs systems (Sivertsen et al. 2012). The mapping of the GLP‐1 receptor localization has been hampered by problems with inadequate specificity of the antibodies employed (Pyke and Knudsen 2013; Pyke et al. 2014; Ussher and Drucker 2014). This is particularly relevant for the cardiovascular system, where a less dense expression of the receptors, compared to the pancreatic islets, makes localization studies more difficult. Recently, the GLP‐1 receptor has been localized to the renal vasculature (Jensen et al. 2015). Moreover, it has been suggested that there may be expression of GLP‐1 receptors in the gastrointestinal system (Pyke et al. 2014), where GLP‐1 has motor functions (Holst 2007) and may promote mucosal growth (Simonsen et al. 2007). These actions are reminiscent of those of the sister peptide, glucagon‐like peptide‐2, produced and secreted in parallel with GLP‐1 from the intestinal L‐cells (Buhl et al. 1988; Hartmann et al. 2000a). Perhaps associated with its growth effects and metabolic actions on the gastrointestinal tract (Bremholm et al. 2011), GLP‐2 markedly stimulates intestinal blood flow in both experimental animals and humans (Bremholm et al. 2009).

We therefore hypothesized that GLP‐1 might have similar actions on mesenteric blood flow in humans. Indeed, GLP‐1 has been demonstrated to dilate precontracted mesenteric arteries (Ban et al. 2008) and to lower vascular resistance in the perfused small intestine in vitro (Hansen and Holst 2002). In vivo, GLP‐1 is metabolized rapidly by the enzyme, dipeptidyl peptidase‐4 (DPP‐4), leading to the formation of a truncated form, GLP‐1_9–36amide_ (Deacon et al. 1995), which is inactive with respect to insulin secretion, but seems to share at least some of its vascular actions with intact GLP‐1 (Ban et al. 2008). For the current studies, we, therefore, also included experiments with the metabolite (and competitive antagonist (Knudsen and Pridal 1996), GLP‐1_9–36 amide_, and with a GLP‐1‐RA, exenatide, which is widely used clinically because of its stability towards DPP‐4 (Klonoff et al. 2008). In this way, we could study effects of the intact hormone as well as its metabolite and a stabilized form, which does not give rise to formation of the antagonist.

It was decided to use subcutaneous (s.c.) injections of rather large doses of all three peptides because this was known to produce a delayed plasma profile temporally reminiscent of meal‐related increases but at augmented levels similar to those observed after, for instance, gastric bypass operations (Vilsbøll et al. 2000; Jacobsen et al. 2012).

## Materials and Methods

### Participants

A total of eight young healthy volunteers (three females), age 24.3 (2.7) [mean (SD)] years, height 176 (10) cm, weight 71 (13) kg, BMI 22.6 (2.1) kg/m^2^ were included. All had been screened by clinical examination and routine blood biochemistry. All participants gave oral and written consent to take part in the study. The Regional Committee on Biomedical Research Ethics (SJ‐339) approved the study protocol, and the trial was carried out in accordance with the Helsinki II declaration and the regulations of the Danish Data Protection Agency. The trial was registered at www.clinicaltrials.gov, Protocol Registration number NCT01988545.

All of the volunteers received s.c. (lower abdomen) GLP‐1, GLP‐1_9–36 amide_, exenatide (Byetta), or isotonic saline (placebo) on four separate trial days, in a double blinded randomized design.

### Test substances

Synthetic human GLP‐1 (99% pure, purchased from Bachem, Bubendorf, Germany) and synthetic human GLP‐1_9–36 amide_ (96% pure, purchased from Bachem, Bubendorf, Germany) were dissolved in saline with 2% albumin (CSL Behring GmbH, Marburg, Germany), sterile filtered, dispensed into glass ampoules, and tested for sterility and pyrogenes. Purity and structure were ascertained by sequence, high‐performance liquid chromatography, and mass analysis. Exenatide (Byetta^R^) and isotonic saline were purchased commercially and used directly.

### Doppler ultrasound scanning

All examinations were performed by the same sonographer (UBA). The Doppler ultrasound equipment was a Vivid E9 (GE Healthcare systems), equipped with a 2.5–4.0 MHz curvilinear transducer. The superior mesenteric artery (SMA), celiac trunk (TC), and renal artery (RA) were examined with participants lying in the supine position and instructed not to move during the trial session.

For each measurement, calculations based on the Doppler spectra were made during five consecutive heartbeats using the auto measure software. All Doppler parameters (time averaged mean velocity [TAMV], diastolic velocity [V_dia_], and systolic velocity [V_sys_]) were calculated by algorithms incorporated in the software of the US apparatus, thus avoiding measurement bias.

Doppler‐US parameters were measured at time −15, 0, 5, 15, 30, 45, 60, 90, and 120 min. The SMA and the TC were examined by a longitudinal epigastric view, and measurements were made as close to the ostia as possible. The right RA was examined by a transverse epigastric view, and measurements were made in the proximal down sloping part of the artery; angle correction was used as necessary. For each time point, we made three measurements in the SMA, TC, and RA.

The volunteers, the laboratory technicians, and the sonographer were all blinded to the sequence of test compounds.

### Calculations

The resistive index (RI) can be used to estimate the resistance in a vessel. In this setup, a falling RI was taken to indicate that the flow was increasing. The RI was calculated as RI = 1 – (*V*_dia_/*V*_sys_) where *V*_dia_ is the maximal diastolic velocity and *V*_sys_ is the maximal systolic velocity. Changes in the TAMV more directly reflect changes of the flow in a vessel.

### Cardiovascular measurements:

Cardiac parameters were continuously measured from time −15 to 120 min. The task force monitor (TFM; CN Systems, Graz, Austria) continuously measures the heart rate, blood pressure (by finger plethysmography), cardiac output (by impedance cardiography), and calculates stroke volume (SV). The continuous, beat‐to‐beat measuring of blood pressure was supplemented with the conventional noninvasive oscillometric blood pressure measurement. All data were stored from time −15 min to 120 min. We used data from the following periods matched with the time measuring points: 0 min: −1–0 min; 5 min: 0–5 min; 15 min: 6–15 min; 25 min: 16–25 min; 35 min: 26–35 min; 45 min: 36–45 min; 60 min: 46–60 min; 75 min: 61–75 min; 90 min: 76–90 min; and 120 min: 91–120 min. This was done to eliminate brief or short‐lasting changes in cardiac parameters due to accidental movement of the volunteer or slight disturbance during scanning.

### Analyses

GLP‐1 (total (i.e., GLP‐1_7–36amide_ + GLP–1_9–36amide_) and intact), exendin‐4, and pancreatic polypeptide (PP) plasma concentrations were measured by radioimmunoassay after ethanol extraction as previously described (Orskov et al. 1994; Kielgast et al. 2011; Dirksen et al. 2013; Wewer Albrechtsen et al. 2015).

Plasma glucose was measured by the glucose oxidase method using a glucose analyzer (Yellow Springs Instrument, YSI Inc., Yellow Springs, OH).

Plasma insulin and C‐peptide levels were measured on a Cobas^®^ 8000, e602 module (Roche Diagnostics GmbH, Mannheim) using, respectively, Cobas Insulin reagents and Cobas Insulin Calibrator, and Cobas C‐Peptide reagents and Cobas C‐Peptide Calibrator. The Cobas module employs a sandwich electro‐chemiluminescense immunoassay (ECLIA).

### Procedure

The experiments were carried out over 4 days under identical circumstances. The participants arrived after an overnight fast, height and weight were measured, and a urine human chorionic gonadotropin (HCG) test was performed on the female volunteers (all tests were negative). Intravenous access was then established. The task force monitor equipment was attached following which the participants rested for 15–20 min in the supine position. Baseline measurements were made twice during the resting period. The following s.c. injections were given in random order on the four experimental days: isotonic saline (1 mL); 10 *μ*g exenatide (1 mL); 1.5 nmol/kg synthetic GLP‐1_9–36 amide_ (1 mL); and 1.5 nmol/kg synthetic GLP‐1 (1 mL).

Venous blood samples were collected into chilled EDTA tubes containing, in final concentrations: valine pyrrolidide (a DPP‐4 inhibitor; 0.01 mmol/L; a gift from Novo Nordisk A/S), and aprotinin (a protease inhibitor; 500 Kallikrein inhibitory unit (KIU)/mL, Trasylol^®^; Bayer, Marburg, Germany) at times −15, 0, 15, 30, 45, 60, 90, and 120 min and centrifuged at 1200*g* for 10 min at 4°C. Plasma was then distributed into ice‐chilled tubes (Minisorb, Nunc, Roskilde, Denmark) and stored at −20°C pending analyses. Serum was stored in cryotubes and stored at −80°C.

### Statistical analysis

Blood flow and cardiac parameters as well as peptide and glucose levels were compared on all days. Data was assessed for normality using the D'Agostino & Pearson omnibus normality test. All data except insulin and c‐peptide followed a normal distribution.

Analyses of normally distributed data were conducted by two‐way ANOVA followed by Tukey post hoc testing if the ANOVA indicated significant differences between the study days. As insulin and C‐peptide data did not follow a normal distribution, we calculated instead the incremental areas under the curve (iAUCs) and compared these using a one‐way ANOVA with a post hoc test (Tukey) if the ANOVA yielded significant differences.

Participant characteristics are expressed as means and standard deviations (SD), while experimental data are expressed as means and standard error of the mean (SEM). Differences resulting in two‐sided *P* < 0.05 were considered significant. Statistical analyses were carried out using Graphpad Prism version 6.00 for Mac, GraphPad Software, La Jolla California USA.

## Results

### Side effects

None of the participants noted any discomfort over the course of the experimental days.

### GLP‐1 and exenatide

**Figure 1:**
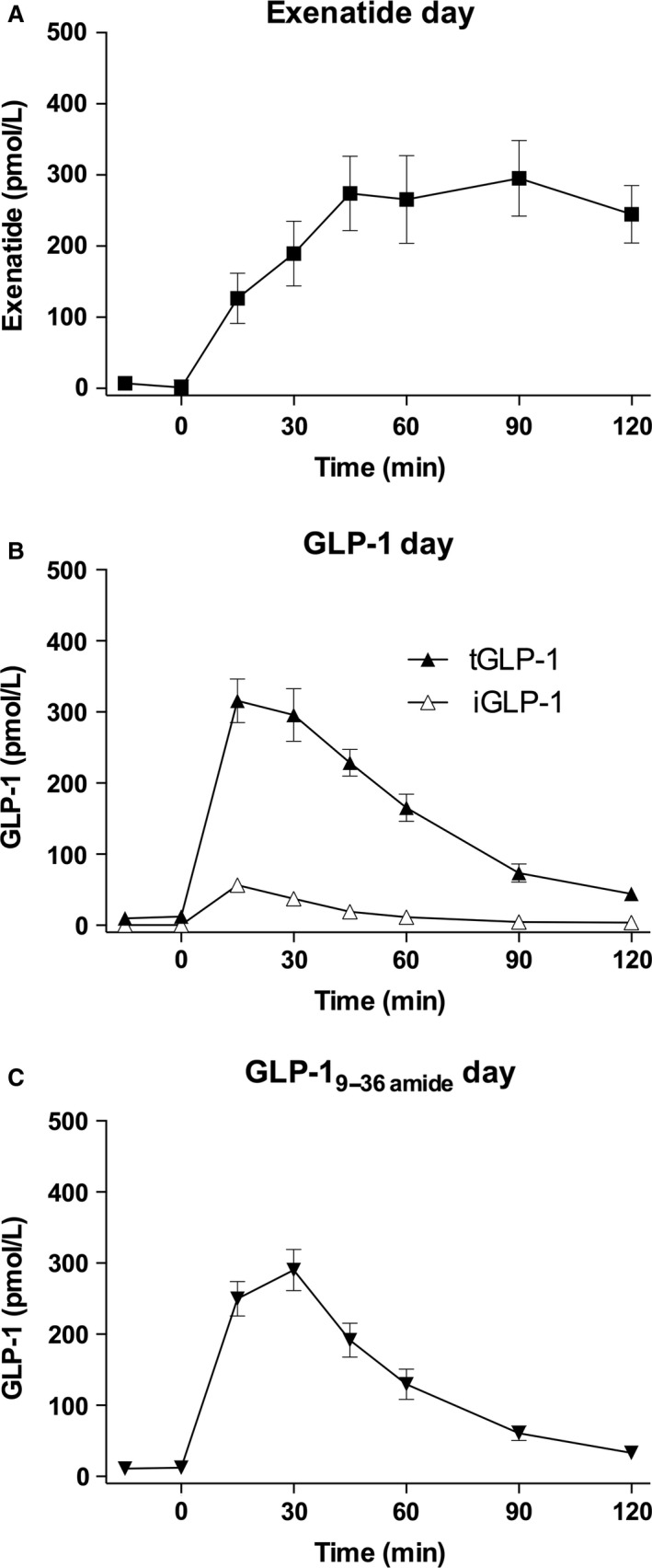
Exendin‐4, GLP‐1, and GLP‐1_9–36amide._ (A) Exendin‐4 levels (squares) on the exenatide day**.** (B) Total (filled triangles) and intact (open triangles) GLP‐1 levels on the GLP‐1 day. (C) Total GLP‐1 levels (inverted triangles) on the GLP‐1_9–36amide_ day. Data are means (SEM).

Plasma concentrations of the test substances are shown in Figure 1. Exenatide levels (Fig. 1A) rose slowly to an apparent plateau around 300 pmol/L at 45 min and did not fall during the observation time. On the GLP‐1 infusion day, total plasma GLP‐1 concentrations (Fig. 1B) rose within 15 min to a mean value around 300 pmol/L and declined slowly thereafter, but were still elevated at 120 min. The levels of intact GLP‐1 increased to approximately 50 pmol/L at 15 min and reached basal levels around 90 min. The concentrations of the metabolite GLP‐1_9–36amide_ (Fig. 1C) rose to about 290 pmol/L at 30 min and declined slowly reaching near basal levels at 120 min.

### Metabolic effects

**Figure 2:**
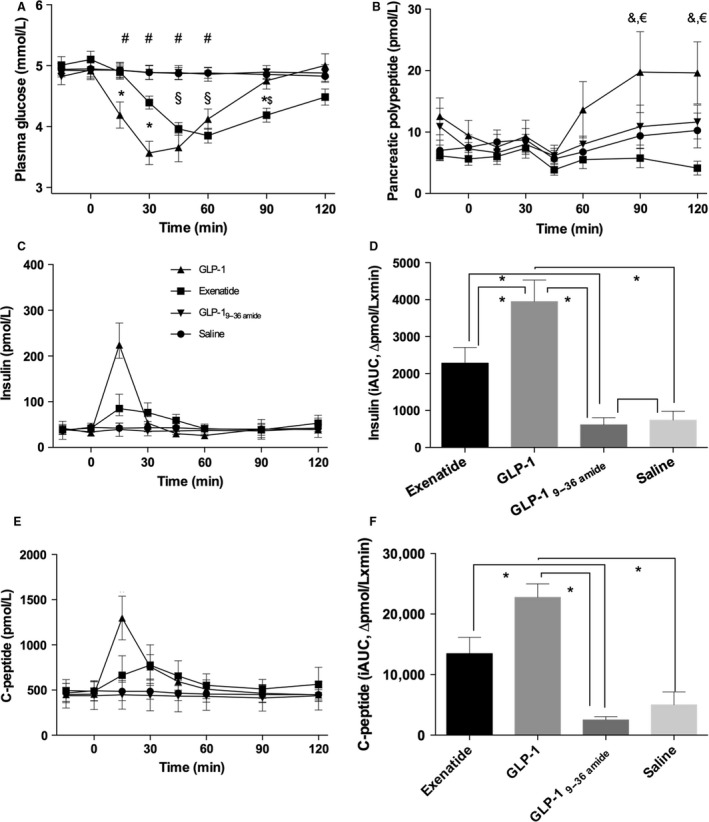
Glucose, insulin, C‐peptide, and pancreatic polypeptide (PP). Symbol legends for the four experimental days; exenatide (squares), GLP‐1 (triangles), GLP‐1_9–36amide_ (inverted triangles), and saline (circles). (A) Plasma glucose: Days were compared by a two‐way ANOVA (infusion (*P* = 0.0001), time (*P* = 0.0001), and infusion × time (*P* = 0.0001)). Post hoc test (Tukey) yielded significant differences; exenatide versus saline/GLP‐1 9–36NH_2_ ($), exenatide versus GLP‐1_7–36amide_ (*), GLP‐1 versus saline/GLP‐1_9–36amide_ (#). (B) Plasma PP: Days were compared by a two‐way ANOVA (infusion (*P* < 0.0001), time (*P* = 0.012), infusion × time (*P* = 0.56)). Post hoc test (Tukey) yielded significant differences; exenatide versus GLP‐1 (&), GLP‐1 versus saline (€). (C) Time courses for serum insulin. (D) Incremental AUCs (iAUCs) for insulin: iAUCs were compared by a one‐way ANOVA (*P* = 0.006), post hoc test (Tukey) yielded significant differences denoted by asterisk (*). (E) Time courses for serum C‐peptide. (F) Incremental AUCs for C‐peptide: iAUCs were compared by a one‐way ANOVA (*P* = 0.0001), post hoc test (Tukey) yielded significant differences denoted by asterix (*). Data are means (SEM). Two‐sided *P* < 0.05 were considered significant.

Plasma glucose (Fig. 2A) levels decreased following GLP‐1 injection from around 5 mmol/L to a nadir of 3.5 mmol/L occurring at 30 min, but returning to basal levels at 90 min. Exenatide also lowered plasma glucose, but less so than GLP‐1 and with a more protracted pattern with a nadir at 60 min. Neither saline nor the metabolite, GLP‐1_9–36amide_ influenced plasma glucose levels.

Insulin (Fig. 2C–D) and C‐peptide (Fig. 2E–F) generally varied in parallel, but with C‐peptide levels being slightly delayed compared to insulin. GLP‐1 caused a rise in insulin, peaking at 15 min and returning to basal levels at 30 min, while C‐peptide levels remained elevated until 60 min. Exenatide also caused a rise in insulin and C‐peptide levels, to about one‐third of that caused by GLP‐1, but peaking around 30 min and thereafter rapidly declining. There were no significant changes in response to GLP‐1_9–36amide_ or saline. In a subset of participants, mean PP levels rose late after the GLP‐1 injection (Fig. 2B) due to excursions in a subset of participants.

### Cardiovascular actions

**Figure 3:**
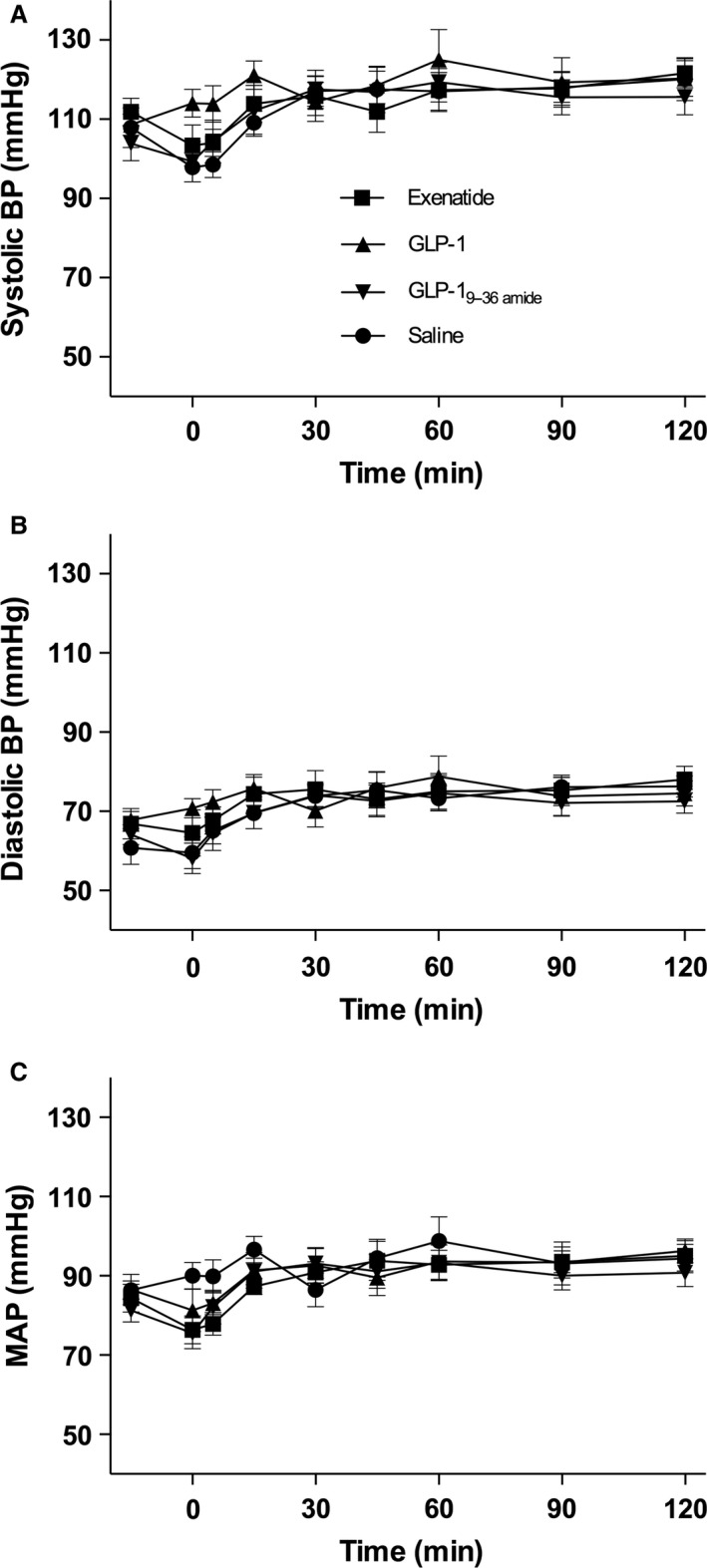
Blood pressures. Symbol legends for the four experimental days; exenatide (squares), GLP‐1 (triangles), GLP‐1_9–36amide_ (inverted triangles), Saline (circles). (A) Systolic blood pressure (Systolic BP): Days were compared by a two‐way ANOVA (infusion (*P* = 0.053), time (*P* < 0.0001), and infusion × time (*P* = 0.93)). (B) Diastolic blood pressure (Diastolic BP): Days were compared by a two‐way ANOVA (infusion (*P* = 0.075), time (*P* < 0.0001), and infusion × time (*P* = 0.99)). (C) Mean arterial blood pressure (MAP): Days were compared by a two‐way ANOVA (infusion (*P* = 0.057), time (*P* < 0.0001), and infusion × time (*P* = 0.92)). Data are means (SEM). Two‐sided *P* < 0.05 were considered significant.

**Figure 4:**
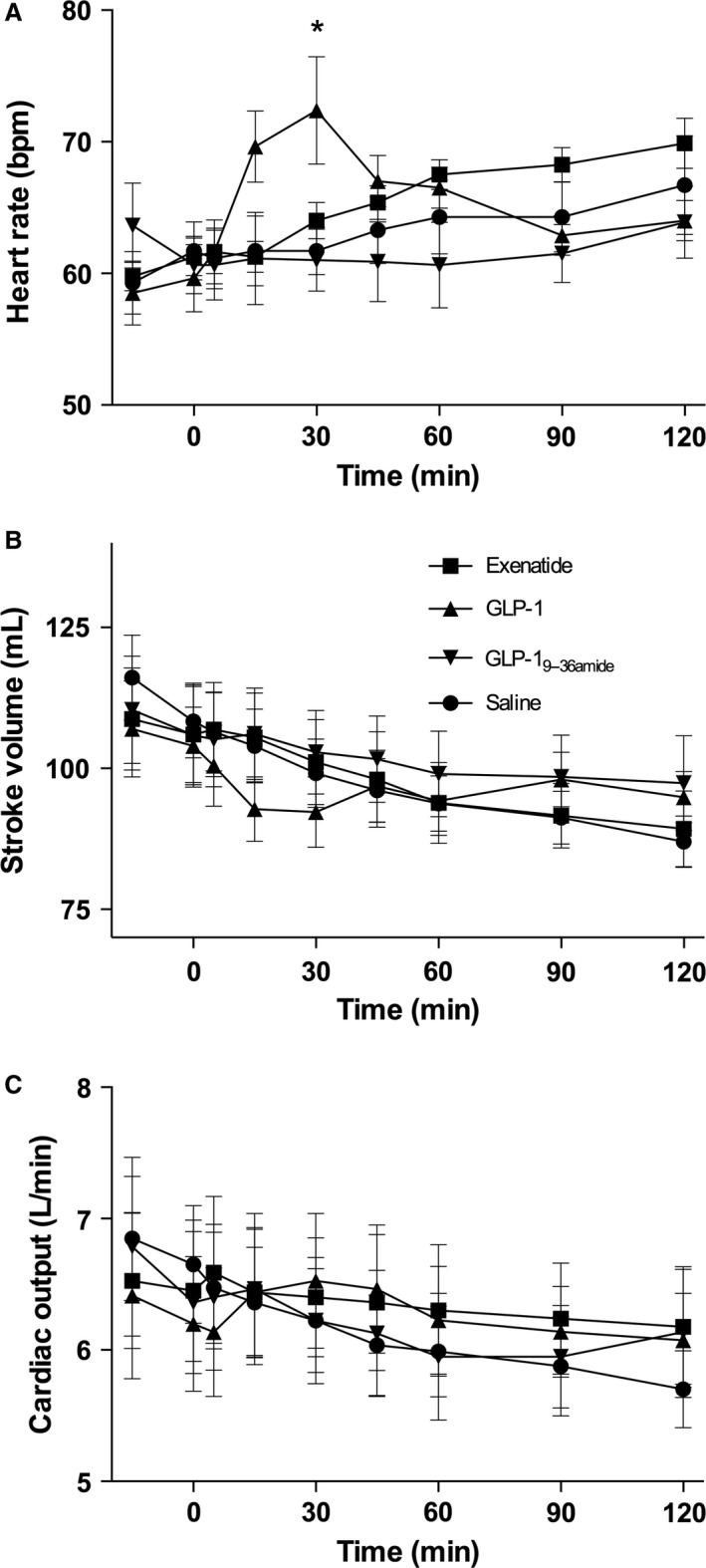
Cardiac parameters. Symbol legends for the four experimental days; exenatide (squares), GLP‐1 (triangles), GLP‐1_9–36amide_ (inverted triangles), Saline (circles). (A) Heart rate: Days were compared by a two‐way ANOVA (injection (*P* = 0.0162), time (*P* = 0.0038), and injection × time (*P* = 0.082)). Post hoc testing (Tukey) yielded a significant difference, GLP‐1 versus saline & GLP‐1_9–36amide_ (*). (B) Stroke volume: Days were compared by a two‐way ANOVA (injection (*P* = 0.51), time (*P* = 0.0050), injection × time (*P* > 0.99)). (C) Cardiac output: Days were compared by a two‐way ANOVA (injection (*P* = 0.92), time (*P* = 0.66), and injection × time (*P* > 0.99)). Data are means (SEM). Two‐sided *P* < 0.05 were considered significant.

Blood pressures are shown in Figure 3 (A–C). The responses were very similar on all days, and there were no significant differences between the groups. Heart rate exhibited different time courses on the infusion days (Fig. 4A). The GLP‐1 injection resulted in an increase by a maximum of about 14 beats per min peaking at 30 min, whereas the injection of exenatide elicited a similar, but slower rise (*P* > 0.05), still rising at 120 min. Heart rate rose slightly after saline but remained stable after GLP‐1_9–36amide_.

Stroke volume (Fig. 4B) decreased similarly (by around 15 mL) in all groups, but appeared to fall more after start of GLP‐1 with lower values at 15 and 30 min, but following the other groups after that. Cardiac output (Fig. 4C) decreased and by about 1 L/min during the 120 min period on the placebo day and a similar pattern was observed after the GLP‐1 metabolite, whereas cardiac output remained stable on the exenatide day, and tended to rise after the GLP‐1 injection in parallel with the rise in heart rate. GLP‐1 briefly increased heart rate (at 15 and 30 min), which was associated with a brief decrease in stroke volume, but nevertheless a small increase in cardiac output.

### Blood flow

**Figure 5:**
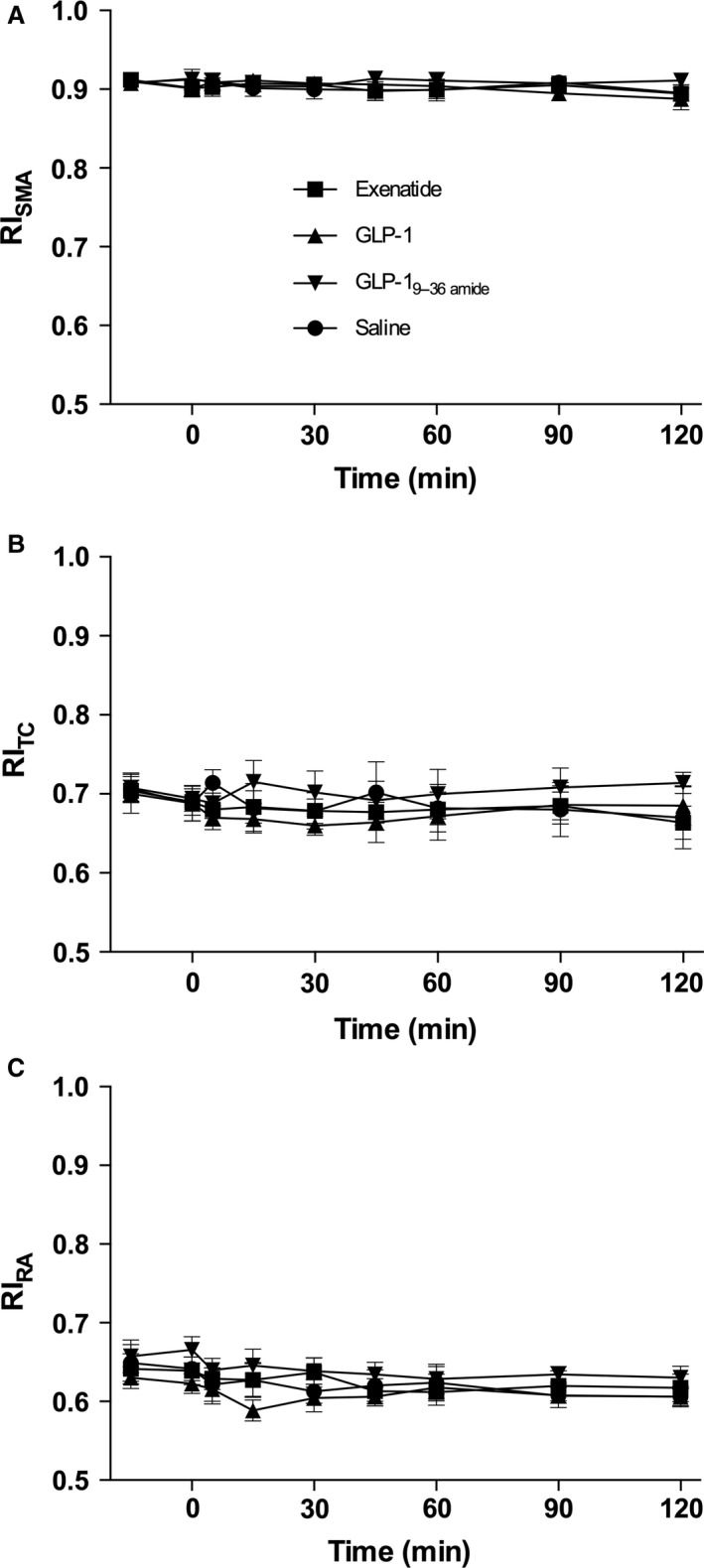
Resistive indices. Symbol legends for the four experimental days; exenatide (squares), GLP‐1 (triangles), GLP‐1_9–36amide_ (inverted triangles), Saline (circles). (A) resistive index (RI) (superior mesenteric artery, SMA): Days were compared by a two‐way ANOVA (infusion (*P *=* *0.45), time (*P *=* *0.85), and infusion × time (*P *>* *0.99)). (B) RI (celiac trunk, TC): Days were compared by a two‐way ANOVA (infusion (*P *=* *0.14), time (*P *=* *0.95), and infusion × time (*P *>* *0.99)). (C) RI (Renal artery, RA): Days were compared by a two‐way ANOVA (infusion (*P* = 0.0044), time (*P* = 0.17), and infusion × time (*P* > 0.99)). Post hoc testing (Tukey) did not yield any time points where curves differed significantly. Data are means (SEM). Two‐sided *P* < 0.05 were considered significant.

**Figure 6:**
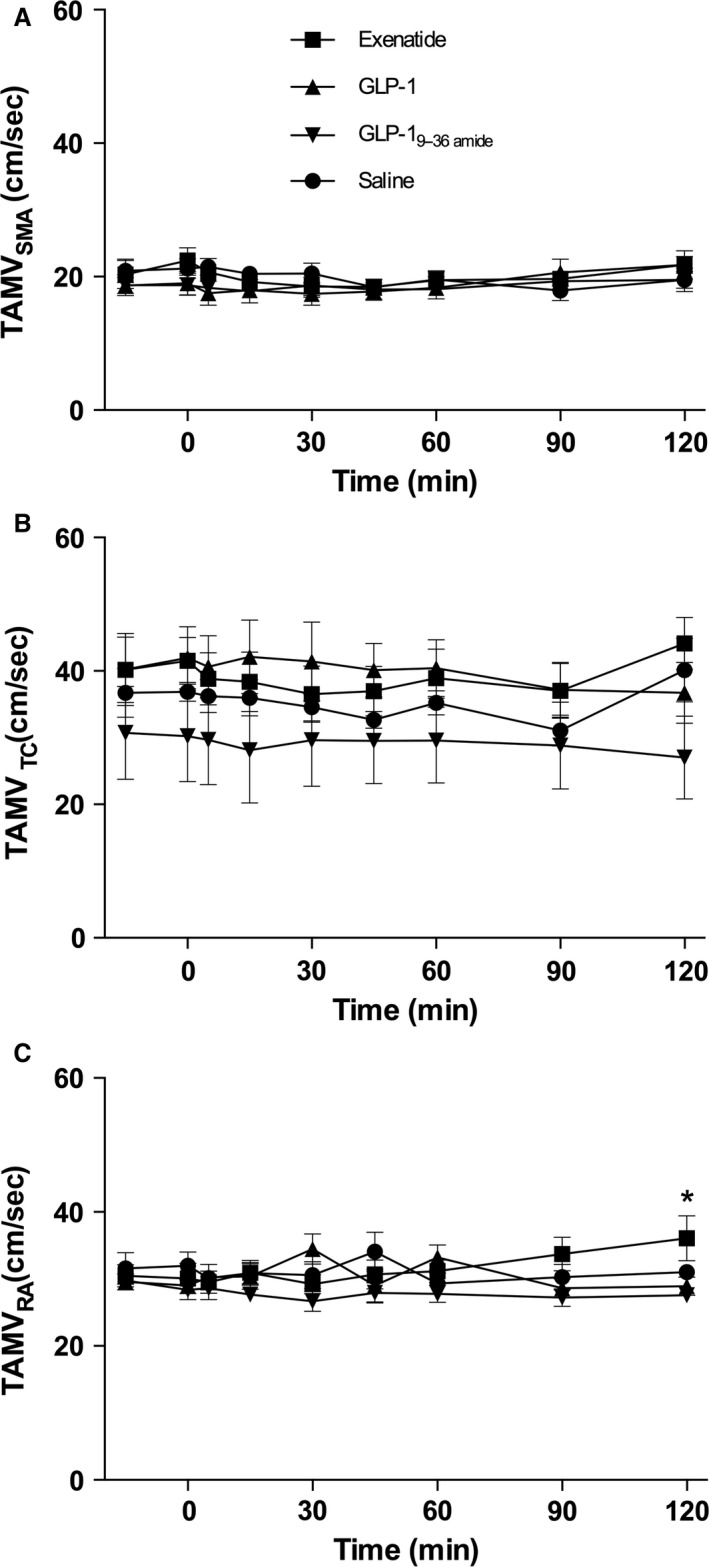
TAMV. Symbol legends for the four experimental days; exenatide (squares), GLP‐1 (triangles), GLP‐1_9–36amide_ (inverted triangles), saline (circles). (A) TAMV (superior mesenteric artery, SMA): Days were compared by a two‐way ANOVA (infusion (*P* = 0.07), time (*P* = 0.36), infusion × time (*P* = 0.97)). (B) TAMV (celiac trunk, TC): Days were compared by a two‐way ANOVA (infusion (*P* < 0.0001), time (*P* = 0.98), and infusion × time (*P* > 0.99)). (C) TAMV (Renal artery, RA): Days were compared by a two‐way ANOVA (infusion (*P* = 0.0003), time (*P* = 0.99), and infusion × time (*P* = 0.35)). Post hoc testing (Tukey) identified a significant difference between exenatide and GLP‐1 and GLP‐1_9–36amide_ (*)**.** Data are means (SEM). Two‐sided *P* < 0.05 were considered significant. TAMV, Time averaged mean velocity.

The results for RI are shown in Figure 5 (A–C) for all three vessels. In the SMA, this parameter remained constant and there were no changes in response to any of the test compounds. The RI in the TC was more variable, but again there were no clear changes after the injections. The RA RI levels differed slightly on the different days and tended to decrease on all days. Also, the TAMV (Fig. 6A–C) showed no consistent changes in the SMA; a more variable pattern was observed in the RA but without consistent changes in response to the injections.

## Discussion

The results regarding blood flow in the superior mesenteric, the celiac and the renal arteries in these studies are highly surprising. As outlined in the introduction, there were compelling reasons to believe that blood flow would increase at least in the SMA. Thus, there are numerous receptors for GLP‐1 in the enteric nervous system, particularly in the enteric ganglia (Pyke et al. 2014), and although the intestinal motor functions of the hormone are probably predominantly inhibitory (Tolessa et al. 1998), GLP‐1 is thought to have metabolic functions and increases the growth of the gut (Simonsen et al. 2007). In addition, administration of GLP‐1 to surviving preparations of the ileum (Hansen and Holst 2002), as well as to isolated mesenteric vessels (Ban et al. 2008), decreases the vascular resistance (Ban et al. 2008), as was originally demonstrated for pulmonary vessels (Richter et al. 1993). The lack of effect is the more striking as the sister hormone, GLP‐2, which is released in parallel with GLP‐1 from the intestinal L‐cells (Orskov et al. 1986) and has similar metabolic actions on the intestine (Drucker et al. 1996; Hartmann et al. 2000b) very markedly stimulates SMA blood flow in healthy humans in an experimental setting identical to the present one (Bremholm et al. 2009). Because the investigators, methods and equipment were the same, we assume that we would have been able to pick up a change in blood flow had there been one. We obtained the plasma concentrations of the test compounds that we had planned, that is, very significant and rather long‐lasting elevations (compared to iv injections) in a meal‐like pattern of both total GLP‐1 and active GLP‐1 concentrations and of the metabolite as well. We deliberately chose a dose of GLP‐1 which would result in higher than normal postprandial concentrations of both the active hormone and the metabolite, because such concentrations may be seen in individuals with accelerated gastric emptying after, e.g., gastric bypass surgery (Korner et al. 2005). Thus, the values obtained are physiologically relevant and at the same time sufficiently high to exclude nondetection of effects because of too weak stimulation. As expected, the injected GLP‐1 was rapidly broken down to the metabolite GLP‐1_9–36amide_, but the concentrations of the intact peptide were elevated for a sufficiently long period to elicit marked effects on insulin and C‐peptide concentrations, and also resulted in significant lowering of blood glucose. Thus, both the injected native GLP‐1 and the stable GLP‐1 receptor agonist exenatide (a synthetic form of exendin‐4, a full GLP‐1 receptor agonist (Thorens et al. 1993) )had the expected metabolic effects demonstrating that the experimental conditions for the experiments were appropriate. In a subset of participants, a marked rise in PP was observed, ostensibly elicited by the drop in the glucose levels, rather than a direct stimulatory effect of GLP‐1(Schwartz et al. 1978)_._ This would account for the timing of the PP increment and also illustrates a probable involvement of the autonomic nervous system at this time point.

Exenatide was included in the study because we wanted to investigate a stable GLP‐1 receptor agonist, which is widely used for therapeutic purposes (Klonoff et al. 2008) and which does *not* give rise to the metabolite, GLP‐1_9–36amide_, which might influence the results. The metabolite was included for the same reason, namely that it might have biological effects of its own. Thus, the metabolite may act as a competitive (but less potent) antagonist on the GLP‐1 receptor (Knudsen and Pridal 1996), which could conceal effects of the intact hormone (Wettergren et al. 1998a); but, in addition, it has been reported to have effects of its own, viz. lowering of plasma glucose levels independently of insulin secretion (Deacon et al. 2002; Meier et al. 2006), and perhaps more importantly to have effects on both the heart and on mesenteric vessels (relaxation) that appear to be independent of the known GLP‐1 receptor (Ban et al. 2008). Neither of these effects could be demonstrated in this study, however.

In agreement with several other studies, both GLP‐1 and exenatide had effects on some of the cardiovascular parameters investigated. The most conspicuous was the increase in heart rate. The increase clearly followed the plasma concentrations of the two peptides with a rapid rise and decline after native GLP‐1 and a steady rise after exenatide. Currently, the mechanisms behind this effect, which is also observed clinically (Sun et al. 2015), are highly debated. However, the mechanisms behind the effects observed chronically during therapy and acutely as in the present study may not be the same. It is currently unclear where the responsible receptor might be located; using improved technology many of the positive findings regarding receptor localization in earlier studies have been refuted (Ussher and Drucker 2014), and the only (surviving) location in the heart of primates appeared to be in the sinoatrial node (Pyke et al. 2014), which could be consistent with a chronotropic effects of GLP‐1 directly on the heart. A similar location could not be identified in rodents, but full‐length transcripts of the receptor were found in atrial tissue (Ussher and Drucker 2014). Acute responses to native GLP‐1 with respect to heart rate in humans have been inconsistent, with most studies showing negative effects (Sivertsen et al. 2012). With the present mode of administration, however, a robust response was obtained which could be helpful in further studies. However, there was also a rather pronounced decrease in blood glucose, which might cause increased central sympathetic outflow. Further investigations with clamping of blood glucose levels would be required to sort this out. Notably, the metabolite did not significantly affect heart rate. At any rate, the increase in heart rate after GLP‐1 was associated with a rise in cardiac output, apparently outweighing the decrease in stroke volume. This may explain the tendency to higher blood pressures during the GLP‐1 infusion, presumably resulting from the effect on heart rate. This is in contrast to observations during chronic GLP‐1 agonist therapy where a drop in blood pressure is regularly observed (Sun et al. 2015). Again, a good explanation for this has not been found, and vasodilation has been proposed to explain this perhaps in combination with natriuretic effects (Gutzwiller et al. 2004), but as documented in the present study, vasodilation in the RA, the SMA, and the TC is unlikely to explain the lower blood pressure, which may rather reflect capillary recruitment in muscles and fat tissue (Sjøberg et al. 2014).

GLP‐1 may have pronounced renal effects including increased natriuresis (Gutzwiller et al. 2006), but the coupling to renal blood flow is uncertain (Asmar et al. 2015), and contrasting results have been reported depending on the experimental setting (Skov et al. 2013; Jensen et al. 2015). We did not measure diuresis in these studies, but on the other hand did not find any changes in renal blood flow in agreement with previous studies in healthy humans (Asmar et al. 2015).

In very recent studies in humans, SMA blood flow was measured during meal intake or intraduodenal glucose instillation (Trahair et al. 2014, 2015). In the first case, SMA blood flow was lowered, whereas in the latter, it increased. The decrease was interpreted to reflect inhibitory effects of GLP‐1 on gastric emptying (Wettergren et al. 1993) (which is probably due to inhibition of central vagal outflow (Wettergren et al. 1998b) which could also influence blood flow), whereas the increase during intraduodenal glucose could have many explanations given the many metabolic reactions resulting from intraduodenal glucose + GLP‐1.

In conclusion, GLP‐1 has metabolic and cardiovascular effects in humans but, surprisingly and unlike its sister peptide GLP‐2, it does not seem to influence intestinal blood flow.

## Conflicts of Interest

The authors have nothing to disclose and no conflicts of interest.
